# Supplementary material for: Confidence in Women’s Health: A Cross Border Survey of Adult Nephrologists
Source: J Clin Med. 2019 Feb 3;8(2):176. doi: 10.3390/jcm8020176 (PMC6406323; doi:10.3390/jcm8020176)
Supplement: Supplementary file 1 [file jcm-08-00176-s001.pdf]

Table S1.: survey form.

March 8, 2018 is both International Women's Day and World Kidney Day. As an adult nephrologist or nephrology trainee, we are interested in your personal experience working with women with chronic kidney disease.

We are trying to gather information about issues facing women with chronic kidney disease and understand variance in patterns of practice. This survey should take less than 10 minutes to complete.

Please answer to the best of your ability.

1. **What is your gender:**
- Male
- Female
- Prefer not to answer

2. **What is your age:**
- Less than 30
- 30-35
- 36-40
- 41-45
- 46-50
- 51-55
- 56-60
- 61-65
- Over 65

3. **How many years have you been in practice?**
- Still in training
- 0-5 years
- 6-10 years
- 11-15 years
- 16-20 years
- More than 20 years

4. **Current Practice Setting:**
- Community
- Academic/University
- Hybrid
- Still in training
- Other (Please Specify)

5. **What is the clinical scope of your practice?**
- Transplant
- General Nephrology
- Dialysis
- Pediatrics
- Still in Training
- Other (Please specify)

6. **What percent of your job is dedicated to clinical care of patients?**
- 0-24%
- 25-49%
- 50-74%
- More than 75%

7. **What country do you work in?**
- United States
- Canada
- Other (Please specify)

8. **What country did you complete your nephrology training in?**
- United States

- Canada
- Other (Please specify)

9. In your program, where are women with kidney disease who are pregnant or planning pregnancy seen (Check ALL that apply)?

- Specialized interdisciplinary kidney disease in pregnancy clinic
- Nephrologist who specializes in pregnancy
- Obstetrical Internal Medicine specialist
- General or Transplant Nephrologist
- Primary care doctor/family doctor
- High Risk Obstetrician or Maternal Fetal Medicine Specialist
- Other clinic/specialist (please specify)

10. Did your nephrology training program include teaching in obstetric nephrology and/or women's health?

- Yes
- No

11. In your training, what type of exposure did you have to obstetrical nephrology and/or women's health? (Check ALL that apply

- Inpatient consults
- Outpatient consults
- Formal lectures
- Dedicated rotation with Obstetrics

12. Approximately how many pregnant women did you see in your nephrology training?

- 0
- 1-5
- 5-10
- 10-15
- 15-20
- >20

13. On Average, how many women of childbearing age have you counseled on family planning or contraception in the last 12 months?

- 0
- Less than one woman per month
- 1-2 women per month
- 3-4 women per month
- 5 or more women per month

14. On average, how many women have you offered pre-conception counselling to in the last 12 months?

- 0
- Less than one woman per month
- 1-2 women per month
- 3-4 women per month
- 5 or more women per month

15. On average, how many pregnant women have you cared for in the last 12 months?

- 0
- Less than one woman per month
- 1-2 women per month

- ☐ 3-4 women per month
- ☐ 5 or more women per month

16. Approximately how many pregnant women on dialysis have you cared for?

- ☐ 0
- ☐ 1-5
- ☐ 5-10
- ☐ 10-15
- ☐ 15-20
- ☐ >20
- ☐ Unknown

17. Do you adjust dialysis intensity for patients who are pregnant?

- ☐ Yes (Please list what regimen you use): __________
- ☐ No
- ☐ I have not managed dialysis in pregnant women

18. How many pregnant women with a kidney transplant have you cared for in the last 5 years?

- ☐ 0
- ☐ 1-5
- ☐ 5-10
- ☐ 10-15
- ☐ 15-20
- ☐ >20
- ☐ I do not manage patients with kidney transplants

19. Have you cared for women under age 18 when they become pregnant?

- ☐ Yes
- ☐ No

20. Please tell us how frequently you document the following when initially evaluating a woman of child bearing age in clinic:

	Never	Rarely	Often	Always
Number of pregnancies				
Number of miscarriages				
Number of terminations				
History of gestational diabetes				
History of gestational hypertension				
History of preeclampsia				
Gestational age of offspring at delivery				
Birth weight of offspring				

21. Please indicate your level of confidence in your ability to care for women with CKD/ESRD in the following situations.

	A) Not at all	B) Somewhat confident	C) Confident	D) Very confident
Manage menstrual disorders				
Counsel on contraception				

Refer for fertility therapy (logistically)				
Counsel on safety of fertility treatment				
Discuss surrogacy for patients who are unable to conceive				
Diagnose and manage menopause				
Diagnose and manage osteoporosis				

22. **Please indicate your level of confidence in your ability to care for pregnant women with CKD/ESRD in the following situations:**

	A) Not at all	B) Somewhat confident	C) Confident	D) Very confident
Counsel on pregnancy outcomes by CKD stage				
Counsel on fetal outcomes by CKD stage				
Counsel on optimal timing of pregnancy in glomerular disease				
Counsel on optimal timing of pregnancy after transplant				
Manage nephrotic syndrome during pregnancy				
Counsel on fetotoxicity of immunosuppression medications				
Manage immunosuppression medications during pregnancy				
Manage immunosuppressive medications during breastfeeding				
Manage dialysis during pregnancy				

23. **Please indicate your level of confidence in your ability to care for pregnant women with CKD/ESRD in the following situations:**

	A) Not at all	B) Somewhat confident	C) Confident	D) Very confident
Counsel on fetotoxicity of antihypertensive medications				
Set appropriate blood pressure goals during pregnancy				

Manage antihypertensive medications during pregnancy				
Diagnose preeclampsia				
Manage antihypertensive medications during breastfeeding				

24. What limits your ability to provide reproductive/obstetric counseling to women with chronic kidney disease (Check ALL that apply)?

- ☐ Lack of training
- ☐ Lack of evidence
- ☐ Lack of clinical guidelines
- ☐ Little personal knowledge and/or confidence in subject matter
- ☐ Lack of time in visit
- ☐ Other (please specify) __________________________

25. As a provider, what resources would be helpful for counseling and management of women of childbearing age with CKD (Check ALL that apply)?

- ☐ Continuing education seminars or case based materials
- ☐ Interdisciplinary guidelines established by Obstetrics and Nephrology
- ☐ Patient education pamphlets or videos with shared decision making tools
- ☐ Referral to a nephrologist with special interest and/or training in women's health
- ☐ Access to e-consultation
- ☐ Other (Please specify)____________________________
